# Association between Birth Region and Time to Tuberculosis Diagnosis among Non–US-Born Persons in the United States

**DOI:** 10.3201/eid2706.203663

**Published:** 2021-06

**Authors:** Amish Talwar, Rongxia Li, Adam J. Langer

**Affiliations:** Centers for Disease Control and Prevention, Atlanta, Georgia, USA

**Keywords:** tuberculosis and other mycobacteria, bacteria, latent tuberculosis, survival analysis, Kaplan-Meier estimate, TB, respiratory infections, proportional hazards models, United States, emigrants, immigrants

## Abstract

Approximately 90% of tuberculosis (TB) cases among non–US-born persons in the United States are attributable to progression of latent TB infection to TB disease. Using survival analysis, we investigated whether birthplace is associated with time to disease progression among non–US-born persons in whom TB disease developed. We derived a Cox regression model comparing differences in time to TB diagnosis after US entry among 19 birth regions, adjusting for sex, birth year, and age at entry. After adjusting for age at entry and birth year, the median time to TB diagnosis was lowest among persons from Middle Africa, 128 months (95% CI 116–146 months) for male persons and 121 months (95% CI 108–136 months) for female persons. We found time to TB diagnosis among non–US-born persons varied by birth region, which represents a prognostic indicator for progression of latent TB infection to TB disease.

Most incident tuberculosis (TB) cases in the United States occur among non–US-born persons (*1*). During 2018, a total of 70.2% of TB disease cases occurred among non–US-born persons, and 46.6% of those cases were diagnosed >10 years after those persons arrived in the United States (*1*). TB disease can occur from recent person-to-person transmission but more commonly is the result of progression of latent TB infection (LTBI) to TB disease. LTBI is a form of TB in which a person is infected with *Mycobacterium tuberculosis*, the causative agent of TB, but remains asymptomatic and noncontagious (*2*). Left untreated, LTBI can progress to TB disease among up to 10% of persons with LTBI within their lifetime (*2*). Among non–US-born persons residing in the United States, >85% of TB disease cases are attributed to progression of LTBI to TB disease (*3*,*4*). Consequently, the Centers for Disease Control and Prevention (CDC) recommends that efforts to eliminate TB in the United States, in part, focus on LTBI detection and treatment among non–US-born persons (*5*).

Understanding the factors associated with LTBI progression can help guide detection and treatment efforts by identifying persons with LTBI who are at greatest risk of developing TB disease and concentrating TB prevention resources toward the populations at highest risk. Although persons recently infected with TB and persons with weakened immune systems are at higher risk for progression to TB disease (*6*), the factors affecting the time to develop TB disease remain unclear, particularly for non–US-born persons. Information on time to develop TB disease can help public health officials target interventions for LTBI testing and treatment among at-risk populations before LTBI progresses to TB disease. One study found the risk of developing TB disease among non–US-born persons decreased with increasing time after entering the United States (*7*). However, non–US-born persons are a heterogenous population who differ in health status based on country of origin (*8*), and the effect of birth country on progression from LTBI to TB disease remains unclear. To clarify disease progression among varying population groups, we evaluated the time to develop TB disease according to birthplace among non–US-born persons with reported cases of TB disease in the United States during 2011–2018.

## Methods

Using national TB surveillance data, we assessed time from entering the United States to TB disease diagnosis among non–US-born persons in whom TB disease developed during 2011–2018. Because of the high number of countries represented among non–US-born persons with TB disease, we categorized birth countries into regions. We assessed time to TB disease diagnosis as time from entry into the United States to time the TB case was reported to a local or state health department. We excluded persons with TB disease attributed to recent transmission (*3*) and focused on persons whose TB disease most likely was caused by progression of LTBI acquired in their birth countries. We compared these times by using bivariate and multivariate survival analysis, and we adjusted for sex, birth year, and age at time of entry into the United States.

### Study Population

We derived the study population for this analysis from CDC’s National Tuberculosis Surveillance System (NTSS), which has been collecting information on TB disease cases from local and state health departments in the United States since 1953 (*9*,*10*). Case reports include demographic, clinical, and risk factor data. The most recent TB surveillance case definition in NTSS, as of 2009, is available in the 2018 US TB surveillance report (*1*). For this study, we examined cases reported to NTSS during January 2011–December 2018 among non–US-born persons, which included persons born outside the United States or its territories for whom neither parent was a US citizen (Appendix).

**Figure 1:**
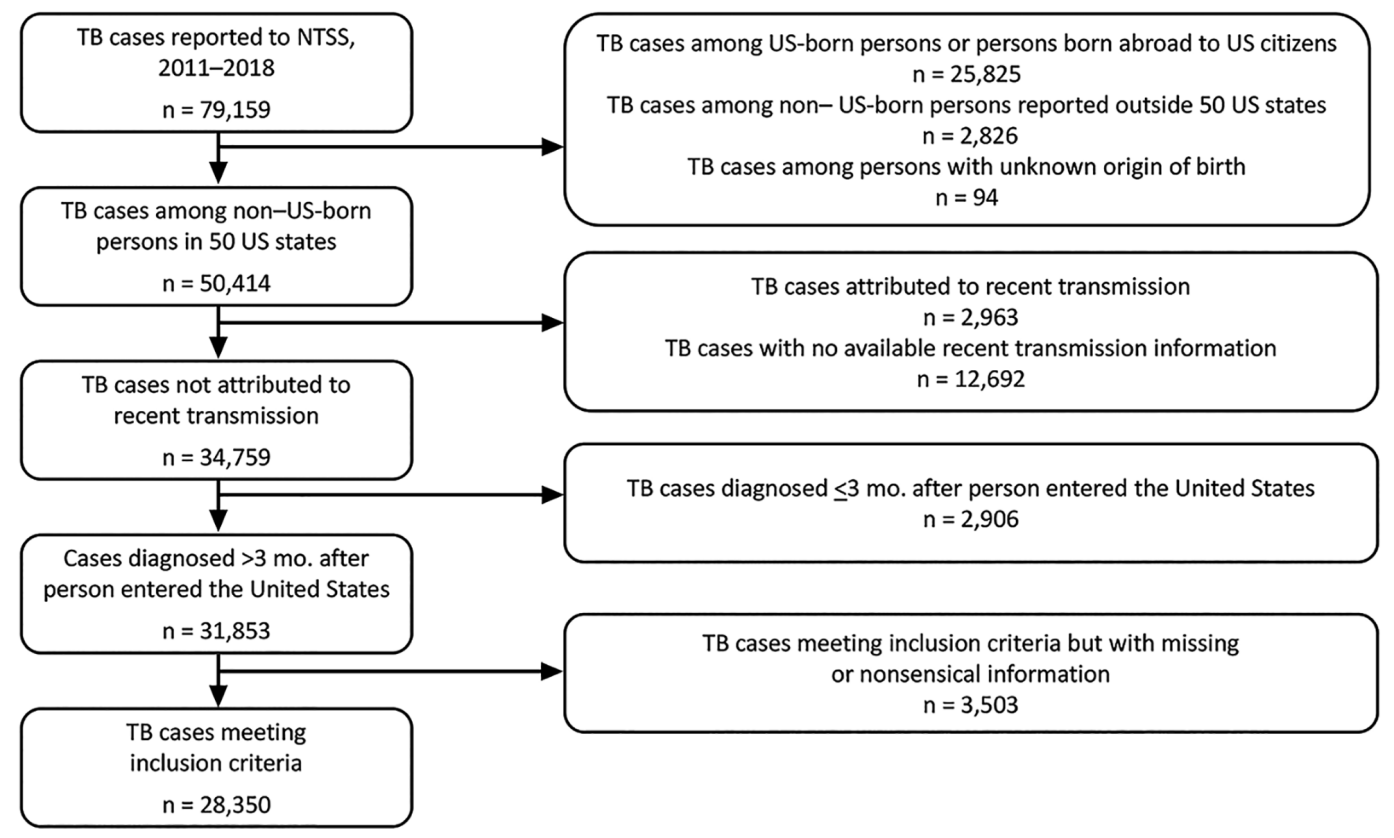
Flowchart of cohort selection process for study evaluating the time to develop TB among non–US-born persons after entering the United States, 2011–2018. NTSS, National Tuberculosis Surveillance System; TB, tuberculosis.

We excluded cases reported from US territories or freely associated states (Figure 1). We also excluded cases attributed to recent TB transmission in the United States by using a previously published method that uses NTSS and genotypic data to determine likelihood of recent transmission (*3*). This plausible-source case method attributes cases to recent transmission if >1 plausible-source case for the case of interest is identified in NTSS (*3*). With this method, cases either are attributed to recent transmission, not attributed to recent transmission, or receive neither designation if they lack the necessary information to assess for recent transmission, such as missing genotype data. By excluding cases attributed to recent transmission, we were able to analyze cases that were more likely the result of LTBI progression. 

We further excluded cases not attributed to recent transmission among persons for whom time from arrival in the United States to TB disease diagnosis was <3 months. We excluded this group because TB disease among persons in the country <3 months can represent disease that was present at time of US entry rather than LTBI reactivation after arrival; excluding these cases also helps account for variability in TB disease screening overseas before entry into the United States. We also excluded cases with missing data and observations with nonsensical values for a category, such as <0 months to TB disease diagnosis, <0 years of age at time of entry, and non–US-born persons with United States listed as birth country.

### Research Design and Variables

We performed a bivariate analysis and a multivariate analysis examining the association between birth country and time from initial arrival in the United States to TB disease diagnosis, which was our main outcome variable. In addition to birth country, we examined the following case demographic variables as additional covariates for our analyses: sex, age at entry in the United States, and birth year. We performed a bivariate analysis to assess the association between time to TB disease diagnosis and these covariates individually, and we performed the multivariate analysis to account for the effects of these covariates on the association between birth country and time to TB disease diagnosis. For our analyses, we defined time to TB disease diagnosis as the number of months spent in the United States before diagnosis, which we derived by subtracting the NTSS-reported month and year of initial entry into the country from the month and year that the TB disease case was reported. We used the case report date because information regarding the actual disease diagnosis date was unavailable through NTSS; the report date represents the earliest notification to a local public health agency that the patient might have TB disease. Because >200 non-US countries and territories of birth were reported to NTSS during the study timeframe, we used the United Nations (UN) standard country or area codes for statistical use, Series M, No. 49 (M49), which categorizes countries and territories according to geographic location and level of development, to divide these countries and territories into 19 regions for ease of statistical analysis (*11*,*12*) (Appendix). To derive age at US entry, we subtracted NTSS-reported year of initial US entry from the year the TB disease case was reported to NTSS and then subtracted this number from NTSS-reported age at diagnosis. For our analyses, we categorized age into 6 categories. We derived birth year by subtracting age from the year the case was reported, and we categorized birth year into 3 categories; we included birth year in our analysis to adjust for a previously observed birth year cohort effect among non–US-born persons with TB disease reported to NTSS (*13*).

### Statistical Analysis

We began by determining the number of patients who had TB disease not attributed to recent transmission and determined the unadjusted median number of months and interquartile range (IQR) that these patients spent in the United States before receiving a TB disease diagnosis. We also determined the unadjusted median number of months according to birth region. We constructed a bivariate Kaplan-Meier survival curve to visually demonstrate the overall distribution of time to TB diagnosis since US entry. We then constructed bivariate Kaplan-Meier curves stratified by each covariate. We tested for statistically significant differences between curves at p<0.05 by using the log rank test and reported global p values.

We then used Cox regression to examine the association of time to TB disease diagnosis and birth region, adjusting for the effects of age at US entry, sex, and birth year. We tested the proportional hazards assumption, and our data met this assumption on the basis of an examination of Schoenfeld residual plots (Appendix). Because our data are likely double-truncated, we applied a correction for double-truncation to our sample set (*14*) (Appendix). Using the Cox regression model, we calculated adjusted median times by birth region by fixing age at US entry to 25–44 years (the category with the most observations for 18/19 birth regions) and birth year at 1940–1979 (the category with the most observations for 14/19 birth regions). We reported adjusted median times and 95% CIs separately by sex. Finally, we stratified the observations by the 6 World Health Organization (WHO) regions to more easily demonstrate interregional variations in time to TB diagnosis, and we repeated the Cox regression analysis accordingly (*15*) (Appendix).

Because the time after US entry at which persons with TB disease typically receive a diagnosis is unclear, previous studies have used a range of times to define cases as disease missed on entry as opposed to LTBI reactivation (*16*–*18*). Therefore, we performed a sensitivity analysis to assess the effect of extending the exclusion window from 3 months to 6 months for TB disease missed at time of entry for our Cox regression analysis.

We performed all statistical analyses by using R version 4.0.2 (R Foundation for Statistical Computing, https://www.r-project.org), and we used the R code developed by Rennert and Xie to correct for double truncation (*14*). All data were collected as part of routine disease surveillance and were not part of human subjects research requiring institutional review board approval.

## Results

During 2011–2018, a total of 79,159 TB cases were reported to NTSS (Figure 1), of which we excluded 28,745 cases because these were among US-born persons, were not reported from 1 of the 50 states or the District of Columbia, or had no known origin of birth reported. We also excluded 15,655 cases that were either attributed to recent transmission or had no information regarding recent transmission available; 2,906 cases for which the time to TB diagnosis was <3 months; and 3,503 cases with missing or nonsensical information for >1 of the variables considered. Because only 5% of all datapoints were missing or nonsensical, and <10% of datapoints were missing or nonsensical for any single variable, we performed listwise deletion of cases with any missing or nonsensical data for our analyses. As a result, we included 28,350 cases for our analyses.

**Figure 2:**
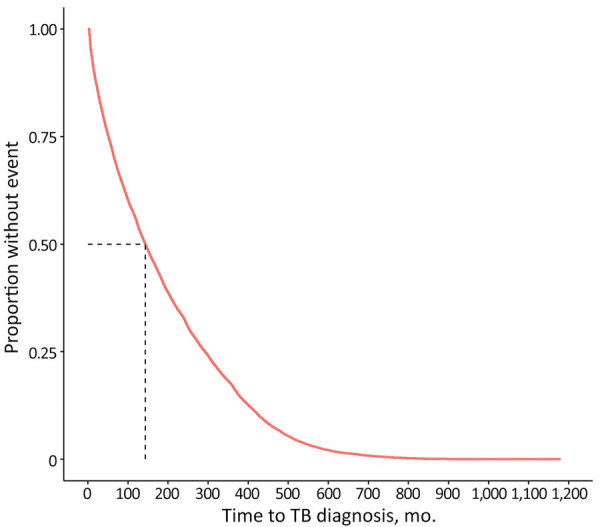
Kaplan-Meier estimate for time to TB disease diagnosis not attributed to recent transmission among non–US-born persons after entering the United States, 2011–2018. Dotted line represents median time for TB disease diagnosis. TB, tuberculosis.

**Figure 3:**
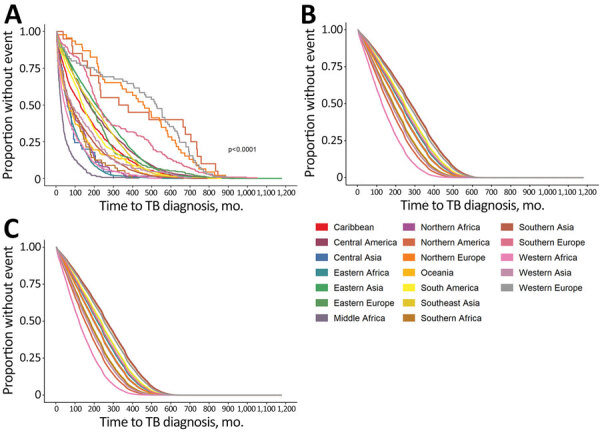
Estimates for time to TB disease diagnosis not attributed to recent transmission among non–US-born persons, stratified by birth region, United States, 2011–2018. A) Kaplan-Meier estimate for all cases; B) Cox regression adjusted time estimates for male patients; C) Cox regression adjusted time estimates for female patients. TB, tuberculosis.

Most non–US-born persons who developed TB disease not attributed to recent transmission after arrival to the United States emigrated from Asia (Table 1); the subregion or intermediary region with the highest proportion of non–US-born persons who received a TB diagnosis in the United States not attributed to recent transmission was South-eastern Asia. The unadjusted median number of months that a non–US-born person who developed TB disease spent in the United States before receiving a TB diagnosis not attributed to recent transmission was 143 months (IQR 51–292 months). Persons from Middle Africa had the lowest unadjusted median number of months until TB diagnosis (26 months), and persons from Western Europe had the highest unadjusted median number of months (524 months) (Table 1). We calculated Kaplan-Meier estimates for time to TB diagnosis unstratified by birth region (Figure 2) and stratified by birth region (Figure 3, panel A). We also calculated Kaplan-Meier estimates for the other covariates, including birth region, sex, age at US entry, and birth year (Appendix Figures 1–3). For all 4 covariates we identified statistically significant differences (p<0.01) in the survival curves.

**Table 1:** Median time to diagnosis of tuberculosis disease not attributed to recent transmission for non–US-born persons by region, United States, 2011–2018

Region	No. (%)	Unadjusted median time, mo (IQR)	Adjusted median time, mo (95% CI)*	
			Male sex	Female sex
Africa	2,900 (10.2)			
Eastern Africa	1,618 (5.7)	61 (25–123)	185 (178–193)	175 (167–183)
Middle Africa	286 (1.0)	26 (11–55)	128 (116–146)	121 (108–136)
Northern Africa	125 (0.4)	62 (25–161)	177 (153–206)	166 (143–194)
Southern Africa	56 (0.2)	77 (28–150)	185 (156–229)	175 (146–215)
Western Africa	815 (2.9)	46 (15–124)	162 (152–174)	152 (142–164)
Americas	9,668 (34.1)			
Caribbean	1,238 (4.4)	127 (45–273)	213 (202–225)	201 (190–212)
Central America	7,071 (24.9)	174 (74–338)	246 (242–250)	236 (229–242)
Northern America†	20 (0.1)	353 (176–713)	264 (223–336)	251 (210–321)
South America	1,339 (4.7)	141 (60–244)	230 (218–241)	216 (206–229)
Asia	14,973 (52.8)			
Central Asia	45 (0.2)	60 (33–97)	187 (158–233)	177 (148–219)
Eastern Asia	2,921 (10.3)	210 (88–363)	277 (266–289)	263 (253–275)
South-eastern Asia	7,793 (27.5)	190 (74–325)	245 (241–248)	235 (229–240)
Southern Asia	4,023 (14.2)	76 (27–193)	210 (203–218)	198 (191–206)
Western Asia	191 (0.7)	83 (27–221)	212 (190–240)	200 (180–228)
Europe	660 (2.3)			
Eastern Europe	328 (1.2)	178 (82–274)	236 (218–251)	222 (206–242)
Northern Europe	46 (0.2)	459 (222–619)	279 (240–343)	265 (228–327)
Southern Europe	221 (0.8)	220 (148–485)	245 (205–308)	236 (193–294)
Western Europe	65 (0.2)	524 (200–645)	276 (244–324)	262 (234–309)
Oceania	149 (0.5)	65 (22–164)	213 (179–262)	201 (168–250)

*Age at arrival fixed at 25–44 y, birth year fixed at 1940–1979. †Excludes United States.

We calculated adjusted median times to TB diagnosis by birth region (Table 1) and adjusted times to TB diagnosis by birth region and sex (Figure 3, panels B, C). We noted persons from Middle Africa had the lowest median adjusted time to TB diagnosis, 128 (95% CI 116–146) months for male sex and 121 (95% CI 108–136) months for female. Persons from Northern Europe had the highest median adjusted time to diagnosis, 279 (95% CI 240–343) months for male sex and 265 (95% CI 228–327) months for female.

**Figure 4:**
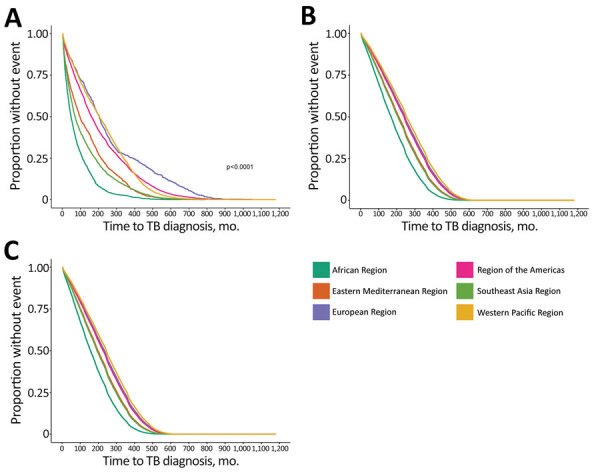
Estimates for time to TB disease diagnosis not attributed to recent transmission for non–US-born persons, stratified by World Health Organization region, United States, 2011–2018. A) Kaplan-Meier estimate for all cases; B) Cox regression adjusted time estimates for male patients; C) Cox regression adjusted time estimates for female patients. TB, tuberculosis.

We also calculated unadjusted and adjusted median times to TB diagnosis by WHO region (Table 2) and the Kaplan-Meier and Cox regression estimates for time to TB diagnosis by WHO region (Figure 4). We found persons from the African Region had the lowest adjusted median time to diagnosis, 169 (95% CI 161–176) months for male sex and 157 (95% CI 150–165) months for female. Finally, after performing our sensitivity analyses, we observed that increasing the exclusion window from <3 months to <6 months changed the adjusted median times for all persons by <10%, regardless of birth region (Appendix Table 4).

**Table 2:** Median time to diagnosis of tuberculosis disease not attributed to recent transmission for non–US-born persons by World Health Organization region, United States, 2011–2018

Region	No. (%)	Unadjusted median time, mo (IQR)	Adjusted median time, mo (95% CI)*	
			Male sex	Female sex
African	2,379 (8.4)	48 (18–111)	169 (161–176)	157 (150–165)
Eastern Mediterranean	1,226 (4.3)	98 (32–215)	211 (202–222)	198 (189–208)
European	779 (2.7)	205 (88–390)	245 (228–266)	234 (214–252)
The Americas†	9,667 (34.1)	161 (67–316)	241 (236–244)	227 (221–234)
Southeast Asian	4,386 (15.5)	71 (26–181)	206 (199–214)	193 (187–201)
Western Pacific	9,913 (35.0)	205 (86–339)	254 (250–260)	244 (240–248)

*Age at arrival fixed at 25–44 y, birth year fixed at 1940–1979. †Excludes United States.

## Discussion

On the basis of our analysis of NTSS data, the median length of time to TB diagnosis varied according to birth region for non–US-born persons in whom TB disease eventually developed. The lowest adjusted median times to TB disease diagnosis were concentrated among persons originating from Africa. In 2018, 5/10 birth countries associated with the highest TB disease rates in the United States were in Africa, and Africa had the highest TB disease rate globally (*1*,*19*). Whether an actual causal association exists between higher overall TB disease rate in one’s birthplace and one’s likelihood of developing TB disease earlier is unclear. However, WHO’s African Region had the highest proportion of LTBI attributable to recent infection in 2014 (*20*), and persons are most likely to develop TB disease soon after TB infection (*5*). Therefore, shorter time to TB disease diagnosis might be associated with higher annual risk for TB infection. Another possibility is that US healthcare providers might have a higher suspicion for TB disease among certain populations, particularly persons from countries with high TB rates, a known risk factor for developing TB disease because of the higher risk for TB infection in those countries (*5*). Certain host genetic factors have been associated with LTBI progression to TB disease (*21*), so a further possibility is that the differences might reflect genetic differences between persons originating from different regions. Although these potential etiologies are compelling, the differences observed in this study simply could be the consequence of overall poorer health status among persons from different regions because of lower levels of economic development and healthcare access. Of note, 33 of the 47 least-developed countries in the world are located in Africa (*22*), and Africa has the lowest overall healthy life expectancy, which is the number of years that a newborn is expected to live in good health (*23*). In addition, persons with poorer health status, particularly malnutrition (*24*), might be more susceptible to developing TB disease than are healthier persons.

The association between birth region and time to TB diagnosis also might be a consequence of differences in risk factors for progression to TB disease among different regions. Both HIV infection and diabetes mellitus are key risk factors for LTBI reactivation, and HIV infection represents the greatest risk factor for LTBI reactivation (*25*). Worldwide, 9% of incident TB cases in 2018 were among persons living with HIV and 15% of TB cases might be linked to diabetes mellitus (*19*,*26*). In addition, the WHO African Region has the highest prevalence of HIV among incident TB cases (*19*), and the HIV diagnosis rates for Africa-born persons living in the United States are 6 times higher than the rate for the general US population (*27*). Given the possibility of TB and HIV co-infection or TB and diabetes mellitus comorbidity as plausible etiologies for the association between birth region and time to TB disease diagnosis, including HIV status or diabetes mellitus diagnosis as variables in our analysis might appear reasonable. However, even though NTSS offers information on HIV status and diabetes mellitus diagnosis, it does not offer information on the date of testing or diagnosis. Therefore, to use these data, we would have to unjustifiably assume that a person’s HIV status or presence of diabetes mellitus were constant throughout the time from US entry to TB disease diagnosis date. This assumption also would be invalid because global HIV incidence was likely low before the 1980s (*28*), and before 2010, HIV infection could prevent non-US citizens from entering the United States (*29*). In addition, including history of diabetes mellitus as a variable in this study would have resulted in an immortal time bias (*30*). For example, persons who received a diagnosis of diabetes mellitus during the study period, before receiving a diagnosis of TB disease, must have persisted long enough without a TB disease diagnosis to receive a diabetes mellitus diagnosis. For these persons, the period of time from the start of the study period to diabetes mellitus diagnosis is known as immortal time because they might not have developed TB disease during that time interval. However, persons who have never received a diagnosis of diabetes mellitus might have received a TB diagnosis during this immortal time, resulting in a disadvantaged survival time despite not having a diabetes mellitus diagnosis. Therefore, we excluded HIV status and diabetes mellitus diagnosis as variables from this study.

Finally, all refugees entering the United States should undergo a domestic medical examination upon entry, unlike most other persons immigrating to the United States (*31*,*32*). Given that Africa and Asia together account for the highest proportion of refugees coming to the United States since 2010 (*32*), their comparatively low adjusted median times in this study might be a consequence of TB disease diagnosis at the time of their domestic medical evaluation, especially since TB screening is a recommended component of that examination (*33*). However, the domestic medical examination is typically conducted 1–3 months after US entry (*34*), which is within our exclusion window for cases attributable to imported TB disease. In addition, all refugees are screened for TB before US entry, making it less likely that newly diagnosed TB would be detected after entering the country.

The first limitation of our study is that the characteristics of persons who immigrate to the United States might not represent persons who remain in their birth country. One study reported that TB incidence in the birth country was 5.4 times higher than the US TB incidence for persons born in those countries and who immigrated to the United States (*35*). Another study found that the prevalence by birth country of isoniazid-resistant and multidrug-resistant TB in the United States better correlated with the prevalence by birth country seen in NTSS data than with the prevalence seen in the birth countries themselves (*36*). This finding suggests that non–US-born persons are not representative of the overall birth country population in terms of TB risk. In addition, NTSS does not report country of immediate origin, which can differ from birth country, nor does NTSS account for interceding travel outside the United States after initial US entry, during which TB might have been transmitted. Finally, the health status of immigrants to the United States might be influenced by long-term US residence, causing it to diverge from expected health status on the basis of birth country.

Our study does not account for the number of non–US-born persons emigrating from a particular region nor how those numbers change over time. We also did not account for reason for immigration, such as refugee status, which might affect risk for TB infection. Furthermore, by using regions to categorize countries in this study, we lose the ability to detect differences between those countries. Also, the plausible-source case method NTSS uses has high accuracy compared with field-based assessments of recent transmission that use epidemiologic investigation methods, but this method is not completely accurate and might misclassify certain cases (*3*). In addition, we were unable to assess the effect of HIV status and diabetes mellitus on time to TB disease diagnosis. Finally, our study did not consider the population of non–US-born persons who did not develop TB disease during the study time period.

In conclusion, time to TB disease diagnosis among non–US-born persons in whom TB disease developed in the United States during 2011–2018 varies by birth region, which represents a prognostic indicator for LTBI progressing to TB disease. Targeted LTBI testing and treatment for persons entering the United States who were born in regions with low median times to TB diagnosis might advance progress toward TB elimination in the United States. Additional studies using data sources that include information on risk factors like HIV infection and diabetes mellitus could help determine the potential influence of these conditions on time to LTBI reactivation; such studies also would benefit from accounting for TB rates in birth countries, where feasible. Similar studies in countries with healthcare systems comparable to the United States and diverse immigrant populations also would help determine whether these results are reproducible and could shed light on the etiology underlying regional differences in time to TB disease diagnosis. Nonetheless, our findings can help focus efforts on LTBI detection and treatment among the most vulnerable populations and further advance efforts to eliminate TB disease in the United States.

Appendix Additional information on evaluation of the time to develop tuberculosis among non–US-born persons after entering the United States, 2011–2018.
