# Supplementary material for: Association between Birth Region and Time to Tuberculosis Diagnosis among Non–US-Born Persons in the United States
Source: Emerg Infect Dis. 2021 Jun;27(6):1645–53. doi: 10.3201/eid2706.203663 (PMC8153865; doi:10.3201/eid2706.203663)
Supplement: Appendix — Additional information on evaluation of the time to develop tuberculosis among non–US-born persons after entering the United States, 2011–2018. [file 20-3663-Techapp-s1.pdf]

Article DOI: https://doi.org/10.3201/eid2706.203663

Association between Birth Region and Time to Tuberculosis Diagnosis among Non–US-Born Persons Entering the United States

Appendix

Birth Country Categorization

We initially categorized each country or territory reported in the National Tuberculosis Surveillance System (NTSS) into 15 subregions and 7 intermediary regions, which are further subdivisions of the Sub-Saharan Africa, Latin America, and Caribbean subregions under the United Nations (UN) standard country or area codes for statistical use, Series M, No. 49 (M49) (*1*). Because of low reported case numbers, we analyzed the subregions of Australia and New Zealand, Melanesia, Micronesia, and Polynesia together under the continental region Oceania, which encompasses these subregions under the M49 standard. As a result, we examined 19 total regions in our analysis. We categorized countries that have changed names but not geographic boundaries, such as Burma/Myanmar and Zaire/Democratic Republic of the Congo, under the region in which the most current country name is categorized. We categorized countries that have changed geographic boundaries, such as Yugoslavia and Czechoslovakia, under the region in which successor countries were categorized. Finally, where the UN does not recognize a country that NTSS reports as a birth country, we assigned that country to a region according to geographic proximity to other countries in that region.

Justification for Cox Regression and Double-Truncation Adjustment

Because we do not have data on the cohort of non–US-born persons who did not develop tuberculosis (TB) disease during the study time period, our Cox regression analysis only examines persons who developed TB, our outcome of interest. As demonstrated by Rennert and Xie (*2*), Cox regression can be used to assess for statistically significant differences among variables of interest while only evaluating persons who experience the outcome of interest (*2*).

However, because this is a retrospective study examining TB cases reported to NTSS from a US jurisdiction, we cannot observe non–US-born persons who developed TB disease before entering the United States (i.e., left truncation), nor can we observe non–US-born persons who will develop TB disease in the future (i.e., right truncation). This phenomenon is known as double-truncation and reflects a sampling bias in which we lack information regarding the entire cohort of non–US-born persons who moved to the United States and at some point develop TB disease. To remedy this sampling bias, we applied a weighted estimation approach that produces weighted estimators of the hazard ratio (*2*). In this approach, the weights are inversely proportional to the probability that a subject is included in the sample (i.e., not truncated), and this probability is conditional on observed survival time. As a result, observations with oversampled survival times are downweighted and observations with undersampled survival times are upweighted (*2*). We used this approach to generate our Cox regression model by using R code sourced from Rennert and Xie (*2*). We calculated percent differences in the β coefficients to compare our model when adjusting for double truncation and our model when not adjusting for double truncation (Appendix Table 1).

Our adjustment for double-truncation was subject to a key limitation: we could not properly test for quasi-independence between truncation times (i.e., time from US entry to study start date and time from US entry to study end date) and event time, which is a necessary precondition for our method of adjustment. Because of the large number of observations examined, we could not correctly assess the p value for the conditional Kendall's Tau used to test quasi-independence. Because no a priori reasons exist to assume dependence between these times, we assumed quasi-independence for the purpose of this analysis. The standard errors of the β coefficients derived when adjusting for double-truncation are smaller than those derived when not making this adjustment, indicating improved prediction accuracy when adjusting for double truncation (Appendix Table 1).

Cox Regression Assumptions

We determined whether the study data met the proportional hazards assumption for Cox regression by generating Schoenfeld residual plots for each demographic covariate, in which we evaluated for independence between residuals and time. A nonrandom pattern against time is evidence of violation of this assumption. Because we observed no such pattern, we determined that the data met the proportional hazards assumption.

References

1. United Nations Statistics Division. Standard country or area codes for statistical use, series M, no. 49 (M49). New York: United Nations; [cited 2020 Mar 2]. https://unstats.un.org/unsd/methodology/m49

2. Rennert L, Xie SX. Cox regression model with doubly truncated data. Biometrics. 2018;74:725–33. PubMed https://doi.org/10.1111/biom.12809

Appendix Table 1. Comparison of β coefficient and standard error estimates of tuberculosis disease onset for non–US-born persons with tuberculosis disease not attributed to recent transmission when adjusting and not adjusting for double truncation, United States, 2011–2018*

	β coefficient			Standard error		
Characteristic	Adjusting for double-truncation	Not adjusting for double-truncation	% difference	Adjusting for double-truncation	Not adjusting for double-truncation	% difference
Age at entry, y						
0–4	−5.03	−2.42	51.9	0.02	0.06	−200.0
5–14	−1.78	−1.64	7.9	0.10	0.03	70.0
15–24	−0.97	−0.83	14.4	0.01	0.02	−100.0
25–44	Referent	Referent	Referent	Referent	Referent	Referent
45–64	1.27	1.07	15.7	0.01	0.02	−100.0
≥65	2.89	2.62	9.3	0.01	0.03	−200.0
Sex						
M	Referent	Referent	Referent	Referent	Referent	Referent
F	0.08	0.08	0.0	0.004	0.01	−150.0
Birth year						
1900–1939	−1.92	−1.69	12.0	0.01	0.02	−100.0
1940–1979	Referent	Referent	Referent	Referent	Referent	Referent
1980–2018	2.75	2.55	7.3	0.01	0.02	−333.3
Region of birth						
Africa						
Eastern Africa	0.64	0.95	−48.4	0.03	0.13	−333.3
Middle Africa	1.12	1.46	−30.4	0.04	0.14	−250.0
Northern Africa	0.71	0.97	−36.6	0.05	0.15	−200.0
Southern Africa	0.64	0.92	−43.8	0.06	0.18	−200.0
Western Africa	0.82	1.10	−34.1	0.03	0.13	−333.3
Americas						
Caribbean	0.43	0.69	−60.5	0.03	0.13	−333.3
Central America	0.20	0.48	−140.0	0.03	0.13	−333.3
Northern America*	0.07	0.12	−71.4	0.07	0.26	−271.4
South America	0.33	0.61	−84.8	0.03	0.13	−333.3
Asia						
Central Asia	0.62	0.88	−41.9	0.06	0.19	−216.7
Eastern Asia	−0.01	0.39	4000.0	0.03	0.13	−333.3
South-eastern Asia	0.21	0.46	−119.0	0.03	0.13	−333.3
Southern Asia	0.45	0.75	−66.7	0.03	0.13	−333.3
Western Asia	0.44	0.74	−68.2	0.04	0.14	−250.0
Europe						
Eastern Europe	0.29	0.54	−86.2	0.04	0.14	−250.0
Northern Europe	−0.02	0.15	850.0	0.05	0.19	−280.0
Southern Europe	0.21	0.28	−33.3	0.04	0.14	−250.0
Western Europe	Referent	Referent	Referent	Referent	Referent	Referent
Oceania	0.43	0.77	−79.1	0.04	0.15	−275.0

*Excludes the United States of America.

Appendix Table 2. Adjusted hazard ratio estimates of tuberculosis disease diagnosis for non–US-born persons with tuberculosis disease not attributed to recent transmission by United Nations region (n = 28,350), United States, 2011–2018*

Characteristic	No. (%)	aHR	95% CI	p value
Age at entry, y				
0–4	270 (1.0)	0.007	(0.001–0.04)	<0.01
5–14	1,567 (5.5)	0.17	(0.15–0.18)	<0.01
15–24	7,565 (26.7)	0.38	(0.36–0.39)	<0.01
25–44	12,062 (42.6)	Referent	Referent	Referent
45–64	5,268 (18.6)	3.58	(3.43–3.73)	<0.01
≥65	1,618 (5.7)	18.00	(16.78–19.30)	<0.01
Sex				
M	16,804 (59.3)	Referent	Referent	Referent
F	11,546 (40.7)	1.09	(1.05–1.12)	<0.01
Birth year				
1900–1939	3,782 (13.3)	0.15	(0.14–0.16)	<0.01
1940–1979	16,180 (57.1)	Referent	Referent	Referent
1980–2018	8,388 (29.6)	15.69	(14.99–16.43)	<0.01
Region of birth				
Africa	2,900 (10.2)			
Eastern Africa	1,618 (5.7)	1.89	(1.47–2.43)	<0.01
Middle Africa	286 (1.0)	3.06	(2.30–4.07)	<0.01
Northern Africa	125 (0.4)	2.02	(1.48–2.78)	<0.01
Southern Africa	56 (0.2)	1.89	(1.32–2.71)	<0.01
Western Africa	815 (2.9)	2.26	(1.75–2.93)	<0.01
Americas	9,668 (34.1)			
Caribbean	1,238 (4.4)	1.54	(1.20–1.99)	<0.01
Central America	7,071 (24.9)	1.23	(0.96–1.57)	0.10
Northern America†	20 (0.1)	1.07	(0.71–1.62)	0.74
South America	1,339 (4.7)	1.39	(1.08–1.79)	<0.05
Asia	14,973 (52.8)			
Central Asia	45 (0.2)	1.85	(1.29–2.65)	<0.01
Eastern Asia	2,921 (10.3)	0.99	(0.77–1.28)	0.96
South-eastern Asia	7,793 (27.5)	1.24	(0.97–1.58)	0.09
Southern Asia	4,023 (14.2)	1.57	(1.23–2.02)	<0.01
Western Asia	191 (0.7)	1.55	(1.16–2.08)	<0.01
Europe	660 (2.3)			
Eastern Europe	328 (1.2)	1.33	(1.02–1.74)	<0.05
Northern Europe	46 (0.2)	0.98	(0.67–1.45)	0.93
Southern Europe	221 (0.8)	1.23	(0.83–1.83)	0.31
Western Europe	65 (0.2)	Referent	Referent	Referent
Oceania	149 (0.5)	1.54	(1.05–2.26)	<0.05
Australia and New Zealand	0 (0.0)	–	–	–
Melanesia	10 (0.04)	–	–	–
Micronesia	110 (0.4)	–	–	–
Polynesia	29 (0.1)	–	–	–

*Countries listed according to United Nations standard country or area codes for statistical use (M49) regions as of January 2020 (*1*). aHR, adjusted hazard ratio; NA, not available; –, not calculated.
†Excludes United States of America.

Appendix Table 3. Adjusted hazard ratio estimates of tuberculosis disease diagnosis for non–US-born persons with tuberculosis disease not attributed to recent transmission, organized by World Health Organization region (n = 28,350), United States, 2011–2018*

Characteristic	No. (%)	aHR	95% CI	p value
Age at entry, y				
0–4	270 (1.0)	0.006	(0.001–0.04)	<0.01
5–14	1,567 (5.5)	0.17	(0.15–0.18)	<0.01
15–24	7,565 (26.7)	0.38	(0.37–0.40)	<0.01
25–44	12,062 (42.6)	Referent	Referent	Referent
45–64	5,268 (18.6)	3.61	(3.46–3.76)	<0.01
≥65	1,618 (5.7)	18.17	(16.95–19.48)	<0.01
Sex				
M	16,804 (59.3)	Referent	Referent	Referent
F	11,546 (40.7)	1.09	(1.06–1.13)	<0.01
Birth year				
1900–1939	3,782 (13.3)	0.14	(0.14–0.15)	<0.01
1940–1979	16,180 (57.1)	Referent	Referent	Referent
1980–2018	8,388 (29.6)	15.59	(14.90–16.32)	<0.01
Region of birth				
African Region	2,379 (8.4)	1.75	(1.52–2.02)	<0.01
Eastern Mediterranean Region	1,226 (4.3)	1.27	(1.09–1.47)	<0.01
European Region	779 (2.7)	Referent	Referent	Referent
Region of the Americas†	9,667 (34.1)	1.04	(0.91–1.20)	0.53
South-East Asian Region	4,386 (15.5)	1.31	(1.14–1.51)	<0.01
Western Pacific Region	9,913 (35.0)	0.93	(0.80–1.07)	0.29

*aHR, adjusted hazard ratio.
†Excludes United States of America.

Appendix Table 4. Comparison of adjusted median times to tuberculosis diagnosis for non–US-born persons with tuberculosis disease not attributed to recent transmission by region according to changes in exclusion criteria, United States, 2011–2018

	Adjusted median time in months for male sex			Adjusted median time in months for female sex		
Region	Original exclusion criteria	Exclude ≤6 mo in United States	% difference	Original exclusion criteria	Exclude ≤6 mo in United States	% difference
Africa						
Eastern Africa	185	187	−1.1	175	178	−1.7
Middle Africa	128	129	−0.8	121	123	−1.7
Northern Africa	177	182	−2.8	166	172	−3.6
Southern Africa	185	193	−4.3	175	183	−4.6
Western Africa	162	166	−2.5	152	156	−2.6
Americas						
Caribbean	213	218	−2.3	201	206	−2.5
Central America	246	248	−0.8	236	240	−1.7
Northern America*	264	267	−1.1	251	254	−1.2
South America	230	235	−2.2	216	221	−2.3
Asia						
Central Asia	187	183	2.1	177	173	2.3
Eastern Asia	277	279	−0.7	263	266	−1.1
South-eastern Asia	245	246	−0.4	235	237	−0.9
Southern Asia	210	215	−2.4	198	203	−2.5
Western Asia	212	218	−2.8	200	206	−3.0
Europe						
Eastern Europe	236	240	−1.7	222	229	−3.2
Northern Europe	279	283	−1.4	265	269	−1.5
Southern Europe	245	247	−0.8	236	239	−1.3
Western Europe	276	280	−1.4	262	267	−1.9
Oceania	213	230	-8.0	201	217	-8.0

*Excludes the United States of America.

Appendix Table 5. Adjusted median times to tuberculosis diagnosis for non–US-born persons from Eastern Africa with tuberculosis disease not attributed to recent transmission, United States, 2011–2018*

Country of birth	No. cases	Unadjusted median time in months (IQR)	Adjusted median time in months for male sex (95% CI)†	Adjusted median time in months for female sex (95% CI)†
Burundi	14	50 (32–120)	109 (62–NA)	101 (58–NA)
Djibouti	4	18 (14–28)	168 (138–211)	156 (128–199)
Eritrea	87	54 (25–107)	160 (142–182)	149 (132–171)
Ethiopia	705	58 (24–111)	163 (136–201)	151 (127–188)
Kenya	255	59 (25–120)	208 (151–339)	195 (140–323)
Madagascar	8	56 (37–68)	290 (258–328)	274 (246–313)
Malawi	11	138 (45–152)	144 (118–190)	134 (109–180)
Mauritius	2	362 (259–464)	295 (280–309)	279 (265–295)
Mozambique	9	35 (28–72)	245 (223–273)	235 (209–258)
Rwanda	14	44 (22–103)	191 (144–284)	181 (134–269)
Somalia	402	77 (29–151)	253 (223–303)	243 (210–287)
South Sudan	1	5 (NA)	232 (187–297)	217 (177–281)
Uganda	20	54 (22–144)	280 (243–340)	265 (231–324)
United Republic of Tanzania	32	74 (22–173)	267 (230–334)	253 (215–318)
Zambia	25	88 (39–144)	427 (422–432)	412 (404–420)
Zimbabwe	29	80 (40–157)	313 (225–577)	299 (211–563)

*IQR, interquartile range; NA, not available.
†Age at arrival fixed at 25–44 y, birth year fixed at 1940–1979.

Appendix Table 6. Adjusted median times to tuberculosis diagnosis for non–US-born persons from Middle Africa with tuberculosis disease not attributed to recent transmission, United States, 2011–2018*

Country of birth	No. cases	Unadjusted median time in months (IQR)	Adjusted median time in months for male sex (95% CI)†	Adjusted median time in months for female sex (95% CI)†
Angola	27	18 (6–39)	179 (169–190)	168 (157–180)
Cameroon	113	30 (18–70)	185 (136–284)	174 (127–269)
Central African Republic	10	22 (15–40)	162 (132–208)	151 (124–195)
Chad	5	31 (17–33)	19 (18–20)	18 (17–18)
Congo	104	22 (9–48)	179 (147–227)	168 (137–213)
Democratic Republic of the Congo‡	19	7 (9–40)	124 (120–129)	117 (111–123)
Equatorial Guinea	4	11 (9–29)	101 (66–230)	94 (62–216)
Gabon	4	42 (31–51)	256 (235–292)	245 (220–277)

*IQR, interquartile range.
†Age at arrival fixed at 25–44 y, birth year fixed at 1940–1979.
‡Zaire was included under the Democratic Republic of the Congo.

Appendix Table 7. Adjusted median times to tuberculosis diagnosis for non–US-born persons from Northern Africa with tuberculosis disease not attributed to recent transmission, United States, 2011–2018*

Country of birth	No. cases	Unadjusted median time in months (IQR)	Adjusted median time in months for male sex (95% CI)†	Adjusted median time in months for female sex (95% CI)†
Algeria	11	58 (33–193)	174 (147–208)	162 (137–195)
Egypt	15	147 (101–243)	144 (105–239)	133 (97–224)
Morocco	30	44 (20–106)	171 (98–NA)	159 (92–NA)
Sudan	66	64 (19–157)	105 (103–107)	98 (95–101)
Tunisia	1	26 (NA)	258 (240–289)	247 (226–273)
Western Sahara	2	317 (298–335)	197 (191–205)	186 (179–193)

*IQR, interquartile range; NA, not available.
†Age at arrival fixed at 25–44 y, birth year fixed at 1940–1979.

Appendix Table 8. Adjusted median times to tuberculosis diagnosis for non–US-born persons from Southern Africa with tuberculosis disease not attributed to recent transmission, United States, 2011–2018*

Country of birth	No. cases	Unadjusted median time in months (IQR)	Adjusted median time in months for male sex (95% CI)†	Adjusted median time in months for female sex (95% CI)†
Botswana	7	156 (146–178)	46 (34–69)	42 (31–64)
Namibia	1	258 (NA)	227 (198–260)	213 (186–248)
South Africa	47	56 (26–125)	246 (179–418)	236 (167–401)
Swaziland	1	15 (NA)	107 (69–252)	99 (64–243)

*IQR, interquartile range; NA, not available.
†Age at arrival fixed at 25–44 y, birth year fixed at 1940–1979.

Appendix Table 9. Adjusted median times to tuberculosis diagnosis for non–US-born persons from Western Africa with tuberculosis disease not attributed to recent transmission, United States, 2011–2018*

Country of birth	No. cases	Unadjusted median time in months (IQR)	Adjusted median time in months for male sex (95% CI)†	Adjusted median time in months for female sex (95% CI)†
Benin	7	27 (10–54)	182 (141–249)	171 (132–240)
Burkina Faso	10	31 (25–63)	135 (108–186)	126 (100–175)
Cabo Verde	18	64 (17–154)	134 (107–185)	125 (99–175)
Côte d'Ivoire	34	37 (16–82)	228 (207–247)	213 (194–238)
Gambia	31	34 (11–79)	422 (416–429)	407 (397–417)
Ghana	69	50 (17–96)	188 (178–201)	177 (167–188)
Guinea	43	33 (11–110)	190 (184–197)	179 (173–185)
Guinea-Bissau	1	14 (NA)	211 (173–270)	198 (161–255)
Liberia	152	62 (15–134)	267 (250–289)	253 (240–273)
Mali	12	112 (57–173)	303 (275–337)	288 (260–321)
Mauritania	9	91 (38–127)	178 (160–199)	167 (149–187)
Niger	6	55 (30–72)	150 (137–166)	139 (128–154)
Nigeria	281	38 (15–110)	227 (211–244)	213 (198–232)
Senegal	40	45 (19–168)	98 (71–164)	92 (66–153)
Sierra Leone	90	57 (21–147)	334 (308–365)	319 (292–353)
Togo	12	58 (19–130)	321 (262–412)	305 (249–395)

*IQR, interquartile range; NA, not available.
†Age at arrival fixed at 25–44 y, birth year fixed at 1940–1979.

Appendix Table 10. Adjusted median times to tuberculosis diagnosis for non–US-born persons from the Caribbean with tuberculosis disease not attributed to recent transmission, United States, 2011–2018*

Country of birth	No. cases	Unadjusted median time in months (IQR)	Adjusted median time in months for male sex (95% CI)†	Adjusted median time in months for female sex (95% CI)†
Antigua and Barbuda	1	558 (NA)	160 (144–179)	149 (134–167)
Bahamas	5	316 (232–514)	187 (125–377)	176 (118–364)
Barbados	1	419 (NA)	254 (190–400)	244 (180–382)
Cuba	131	251 (110–455)	144 (119–188)	134 (110–177)
Dominica	1	273 (NA)	136 (108–190)	127 (100–180)
Dominican Republic	267	119 (40–252)	126 (122–132)	119 (115–124)
Grenada	3	349 (177–432)	176 (110–490)	165 (102–479)
Haiti	746	108 (38–232)	199 (187–213)	187 (176–201)
Jamaica	48	173 (64–329)	205 (164–272)	192 (153–258)
Saint Lucia	2	160 (143–176)	277 (260–296)	261 (248–278)
Trinidad and Tobago	32	178 (114–449)	216 (164–321)	204 (153–306)
Turks and Caicos Islands	1	62 (NA)	254 (190–400)	244 (180–382)

*IQR, interquartile range; NA, not available.
†Age at arrival fixed at 25–44 y, birth year fixed at 1940–1979.

Appendix Table 11. Adjusted median times to tuberculosis diagnosis for non–US-born persons from Central America with tuberculosis disease not attributed to recent transmission, United States, 2011–2018*

Country of birth	No. cases	Unadjusted median time in months (IQR)	Adjusted median time in months for male sex (95% CI)†	Adjusted median time in months for female sex (95% CI)†
Belize	15	394 (176–503)	137 (105–203)	127 (98–190)
Costa Rica	9	268 (150–370)	151 (101–319)	141 (94–304)
El Salvador	408	144 (63–267)	191 (126–416)	181 (119–399)
Guatemala	868	77 (33–142)	304 (248–398)	289 (237–381)
Honduras	563	86 (33–153)	191 (183–202)	180 (171–190)
Mexico	5,110	214 (105–379)	196 (162–248)	185 (151–239)
Nicaragua	82	274 (129–360)	263 (238–304)	249 (223–288)
Panama	16	191 (99–384)	209 (148–360)	196 (138–345)

*IQR, interquartile range.
†Age at arrival fixed at 25–44 y, birth year fixed at 1940–1979.

Appendix Table 12. Adjusted median times to tuberculosis diagnosis for non–US-born persons from Northern America with tuberculosis disease not attributed to recent transmission, United States, 2011–2018*

Country of birth	No. cases	Unadjusted median time in months (IQR)	Adjusted median time in months for male sex (95% CI)†	Adjusted median time in months for female sex (95% CI)†
Canada	20	353 (176–713)	241 (190–325)	228 (180–310)

*IQR, interquartile range.
†Age at arrival fixed at 25–44 y, birth year fixed at 1940–1979.

Appendix Table 13. Adjusted median times to tuberculosis diagnosis for non–US-born persons from South America with tuberculosis disease not attributed to recent transmission, United States, 2011–2018*

Country of birth	No. cases	Unadjusted median time in months (IQR)	Adjusted median time in months for male sex (95% CI)†	Adjusted median time in months for female sex (95% CI)†
Argentina	27	216 (155–314)	110 (68–313)	101 (63–299)
Plurinational State of Bolivia	80	218 (125, 349)	145 (124–178)	135 (117–166)
Brazil	95	122 (54–214)	88 (64–143)	82 (60–134)
Chile	9	336 (247–480)	262 (229–320)	249 (214–305)
Colombia	203	161 (65–310)	208 (205–211)	195 (190–201)
Ecuador	340	126 (54–204)	162 (143–186)	151 (133–175)
Guyana	72	165 (44–262)	248 (197–339)	238 (186–324)
Paraguay	3	199 (104–264)	368 (318–448)	357 (303–432)
Peru	443	130 (64–216)	299 (219–497)	283 (206–484)
Suriname	1	187 (NA)	301 (205–643)	285 (193–630)
Uruguay	10	178 (120–290)	207 (144–374)	194 (133–363)
Bolivarian Republic of Venezuela	56	101 (31–231)	176 (144–223)	164 (134–209)

*IQR, interquartile range; NA, not available.
†Age at arrival fixed at 25–44 y, birth year fixed at 1940–1979.

Appendix Table 14. Adjusted median times to tuberculosis diagnosis for non–US-born persons from Central Asia with tuberculosis disease not attributed to recent transmission, United States, 2011–2018*

Country of birth	No. cases	Unadjusted median time in months (IQR)	Adjusted median time in months for male sex (95% CI)†	Adjusted median time in months for female sex (95% CI)†
Kazakhstan	7	41 (16–158)	17 (7–NA)	15 (7–NA)
Kyrgyzstan	14	42 (35–59)	103 (99–109)	96 (91–101)
Tajikistan*	1	12 (NA)	254 (190–400)	244 (180–382)
Turkmenistan	3	68 (47–137)	188 (156–242)	178 (145–229)
Uzbekistan	20	83 (60–131)	148 (121–194)	138 (113–184)

*IQR, interquartile range; NA, not available.
†Age at arrival fixed at 25–44 y, birth year fixed at 1940–1979.

Appendix Table 15. Adjusted median times to tuberculosis diagnosis for non–US-born persons from Eastern Asia with tuberculosis disease not attributed to recent transmission, United States, 2011–2018*

Country of birth	No. cases	Unadjusted median time in months (IQR)	Adjusted median time in months for male sex (95% CI)†	Adjusted median time in months for female sex (95% CI)†
China	1,847	170 (68–306)	183 (135–280)	172 (126–265)
China, Hong Kong Special Administrative Region	100	321 (226–433)	182 (170–196)	171 (158–185)
China, Macao Special Administrative Region	4	311 (186–439)	247 (224–276)	236 (210–261)
Democratic People’s Republic of Korea	162	296 (148–412)	188 (156–242)	178 (145–229)
Japan	60	377 (181–518)	172 (100–NA)	160 (93–NA)
Mongolia	45	47 (20–76)	252 (247–259)	242 (237–247)
Republic of Korea	543	305 (155–411)	95 (62–217)	88 (58–204)
Taiwan (Republic of China)	160	366 (214–444)	210 (134–527)	198 (125–512)

*IQR, interquartile range; NA, not available.
†Age at arrival fixed at 25–44 y, birth year fixed at 1940–1979.

Appendix Table 16. Adjusted median times to tuberculosis diagnosis for non–US-born persons from Southeast Asia with tuberculosis disease not attributed to recent transmission, United States, 2011–2018*

Country of birth	No. cases	Unadjusted median time in months (IQR)	Adjusted median time in months for male sex (95% CI)†	Adjusted median time in months for female sex (95% CI)†
Cambodia	364	334 (155–385)	211 (130–656)	199 (123–640)
Indonesia	184	96 (27–177)	272 (231–346)	257 (216–329)
Lao People’s Democratic Republic	352	327 (233–406)	180 (116–436)	169 (108–421)
Malaysia	40	137 (30–224)	289 (272–308)	274 (256–293)
Myanmar‡	419	54 (25–95)	236 (229–242)	222 (214–230)
Philippines	3,841	187 (85–327)	93 (43–NA)	86 (40–NA)
Singapore	5	161 (94–195)	224 (179–301)	210 (168–285)
Thailand	184	116 (36–282)	189 (158–240)	179 (147–225)
Viet Nam	2,404	212 (77–311)	182 (176–187)	171 (163–178)

*IQR, interquartile range; NA, not available.
†Age at arrival fixed at 25–44 y, birth year fixed at 1940–1979.
‡Burma included under Myanmar

Appendix Table 17. Adjusted median times to tuberculosis diagnosis for non–US-born persons from Southern Asia with tuberculosis disease not attributed to recent transmission, United States, 2011–2018*

Country of birth	No. cases	Unadjusted median time in months (IQR)	Adjusted median time in months for male sex (95% CI)†	Adjusted median time in months for female sex (95% CI)†
Afghanistan	119	156 (30–324)	125 (93–193)	117 (86–183)
Bangladesh	248	63 (23–158)	192 (180–207)	181 (169–194)
Bhutan	174	43 (18–64)	90 (64–156)	84 (60–145)
India	2,630	85 (27–201)	275 (214–395)	259 (202–378)
Islamic Republic of Iran	74	246 (154–396)	251 (197–360)	241 (186–345)
Nepal	369	42 (21–76)	158 (138–183)	147 (129–172)
Pakistan	393	155 (44–295)	229 (181–310)	215 (170–296)
Sri Lanka	16	91 (58–159)	232 (200–268)	217 (188–253)

*IQR, interquartile range.
†Age at arrival fixed at 25–44 y, birth year fixed at 1940–1979.

Appendix Table 18. Adjusted median times to tuberculosis diagnosis for non–US-born persons from Western Asia with tuberculosis disease not attributed to recent transmission, United States, 2011–2018*

Country of birth	No. cases	Unadjusted median time in months (IQR)	Adjusted median time in months for male sex (95% CI)†	Adjusted median time in months for female sex (95% CI)†
Armenia	15	199 (159–258)	181 (126–322)	171 (119–307)
Azerbaijan	14	56 (23–224)	184 (127–330)	173 (120–314)
Georgia	18	108 (37–178)	216 (193–243)	203 (182–232)
Iraq	33	63 (16–194)	230 (203–259)	216 (190–247)
Israel	6	429 (376–577)	283 (213–447)	268 (200–432)
Jordan	3	49 (27–315)	215 (199–235)	202 (187–221)
Kuwait	2	44 (24–63)	196 (147–295)	185 (137–279)
Lebanon	6	272 (235–335)	279 (264–297)	264 (250–281)
Qatar	2	17 (14–19)	195 (129–415)	185 (122–398)
Saudi Arabia	28	33 (9–49)	201 (150–305)	188 (139–290)
Syria	8	54 (27–107)	473 (409–562)	456 (391–548)
Turkey	19	169 (131–289)	415 (398–430)	397 (382–414)
United Arab Emirates	6	39 (11–126)	208 (99–NA)	195 (93–NA)
Yemen	31	81 (16–217)	199 (191–207)	187 (182–193)

*IQR, interquartile range; NA, not available.
†Age at arrival fixed at 25–44 y, birth year fixed at 1940–1979.

Appendix Table 19. Adjusted median times to tuberculosis diagnosis for non–US-born persons from Eastern Europe with tuberculosis disease not attributed to recent transmission, United States, 2011–2018*

Country of birth	No. cases	Unadjusted median time in months (IQR)	Adjusted median time in months for male sex (95% CI)†	Adjusted median time in months for female sex (95% CI)†
Belarus	5	161 (128–261)	161 (115–280)	151 (106–266)
Bulgaria	11	79 (40–165)	104 (89–127)	97 (82–120)
Czechia‡	2	–	–	–
Czechoslovakia	1	676 (526–756)	141 (116–185)	131 (107–175)
Hungary	10	492 (283–663)	259 (253–266)	247 (242–253)
Poland	55	249 (148–369)	23 (16–40)	21 (14–37)
Republic of Moldova	13	77 (31–132)	179 (163–196)	168 (151–186)
Romania	45	197 (81–306)	252 (223–300)	242 (209–284)
Russian Federation	88	163 (82–268)	72 (41–434)	67 (38–419)
Ukraine	98	168 (71–238)	429 (371–511)	415 (360–497)

*IQR, interquartile range; –, not calculated.
†Age at arrival fixed at 25–44 y, birth year fixed at 1940–1979.
‡Subsumed under the Czechoslovakia because former constituent state of Czechoslovakia.

Appendix Table 20. Adjusted median times to tuberculosis diagnosis for non–US-born persons from Northern Europe with tuberculosis disease not attributed to recent transmission, United States, 2011–2018*

Country of birth	No. cases	Unadjusted median time in months (IQR)	Adjusted median time in months for male sex (95% CI)†	Adjusted median time in months for female sex (95% CI)†
Denmark	1	734 (NA)	119 (94–161)	111 (87–150)
Estonia	1	139 (NA)	156 (126–204)	145 (119–191)
Finland	3	179 (117–381)	310 (226–548)	296 (212–534)
Iceland	2	212 (148–275)	247 (223–278)	237 (210–263)
Ireland	6	599 (556–636)	246 (199–325)	235 (187–310)
Latvia	3	746 (601–787)	196 (161–250)	185 (150–240)
Lithuania	4	219 (195–225)	170 (130–240)	158 (123–226)
Norway	1	5 (NA)	158 (112–281)	147 (103–266)
United Kingdom of Great Britain and Northern Ireland	25	487 (346–605)	67 (40–240)	62 (37–225)

*IQR, interquartile range; NA, not available.
†Age at arrival fixed at 25–44 y, birth year fixed at 1940–1979.

Appendix Table 21. Adjusted median times to tuberculosis diagnosis for non–US-born persons from Southern Europe with tuberculosis disease not attributed to recent transmission, United States, 2011–2018*

Country of birth	No. cases	Unadjusted median time in months (IQR)	Adjusted median time in months for male sex (95% CI)†	Adjusted median time in months for female sex (95% CI)†
Albania	32	151 (61–195)	152 (83–NA)	142 (76–NA)
Bosnia and Herzegovina‡	60	–	–	–
Croatia‡	8	–	–	–
Greece	19	508 (391–623)	243 (201–306)	231 (188–291)
Italy	36	612 (378–726)	248 (229–277)	239 (214–262)
Montenegro‡	3	–	–	–
North Macedonia‡	2	–	–	–
Portugal	20	366 (285–484)	157 (124–215)	146 (116–202)
Republic of Kosovo‡	1	–	–	–
Serbia‡	13	–	–	–
Slovenia‡	4	–	–	–
Spain	15	82 (43–191)	235 (202–272)	220 (189–257)
Yugoslavia	8	193 (156–255)	435 (401–479)	420 (384–462)

*IQR, interquartile range; NA, not available; –, not calculated.
†Age at arrival fixed at 25–44 y, birth year fixed at 1940–1979.
‡Subsumed under Yugoslavia because former constituent state of Yugoslavia.

Appendix Table 22. Adjusted median times to tuberculosis diagnosis for non–US-born persons from Western Europe with tuberculosis disease not attributed to recent transmission, United States, 2011–2018*

Country of Birth	No. cases	Unadjusted median time in months (IQR)	Adjusted median time in months for male sex (95% CI)†	Adjusted median time in months for female sex (95% CI)†
Austria	1	715 (NA)	176 (126, 288)	165 (119, 273)
Belgium	2	307 (175–439)	147 (119–199)	137 (110–187)
France	18	206 (26–456)	208 (202–215)	196 (189–204)
Germany	40	577 (421–683)	126 (86–250)	119 (79–241)
Netherlands	2	292 (175–408)	214 (205–223)	201 (192–210)
Switzerland	2	461 (433–489)	252 (208–331)	242 (195–315)

*IQR, interquartile range; NA, not available.
†Age at arrival fixed at 25–44 y, birth year fixed at 1940–1979.

Appendix Table 23. Adjusted median times to tuberculosis diagnosis for non–US-born persons from Oceania with tuberculosis disease not attributed to recent transmission, United States, 2011–2018*

Country of birth	No. cases	Unadjusted median time in months (IQR)	Adjusted median time in months for male sex (95% CI)†	Adjusted median time in months for female sex (95% CI)†
Cook Islands	4	10 (9–23)	135 (98–230)	126 (91–216)
Fiji	7	100 (53–297)	234 (218–247)	219 (206–235)
Marshall Islands	67	48 (12–133)	251 (242–265)	241 (230–251)
Federated States of Micronesia	41	70 (31–122)	156 (111–269)	145 (102–254)
Nauru	1	63 (NA)	177 (155–204)	166 (145–192)
Palau	1	70 (NA)	210 (158–321)	198 (147–305)
Papua New Guinea	3	6 (6–17)	152 (123–205)	142 (115–193)
Pitcairn	1	388 (NA)	291 (228–422)	276 (213–407)
Samoa	4	409 (307–494)	25 (10–NA)	22 (10–NA)
Tokelau	1	488 (NA)	221 (149–444)	208 (139–427)
Tonga	19	368 (131–412)	344 (283–439)	328 (268–424)

*IQR, interquartile range; NA, not available.
†Age at arrival fixed at 25–44 y, birth year fixed at 1940–1979.

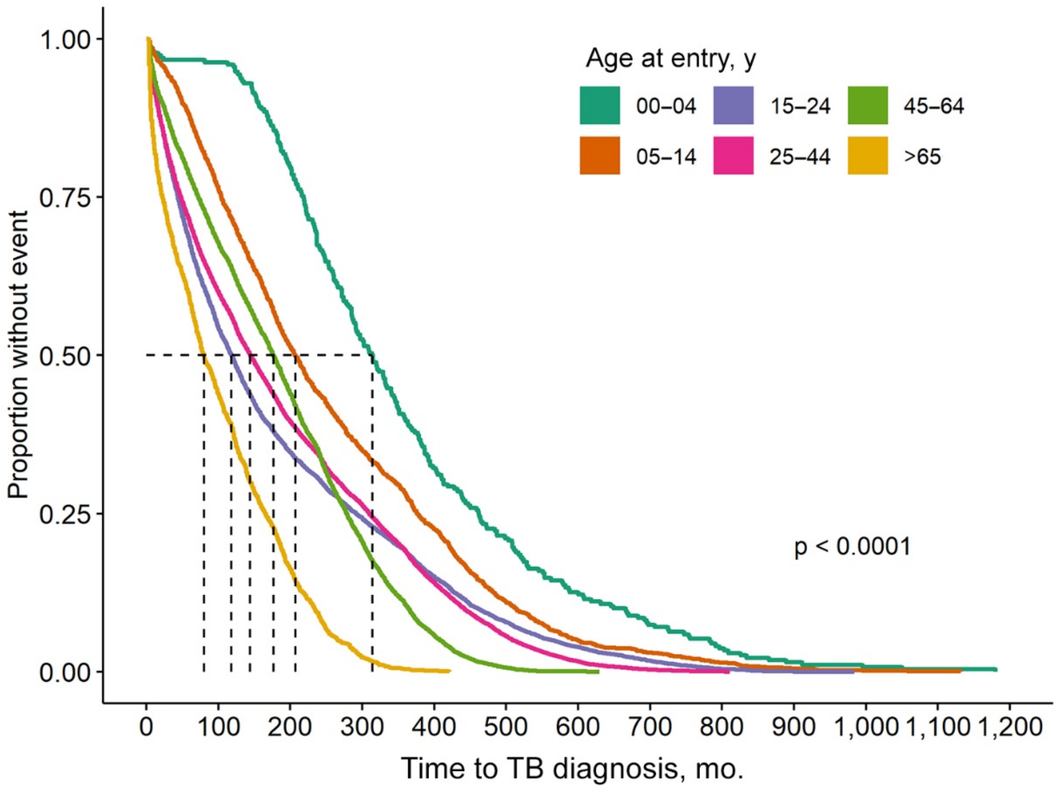

Appendix Figure 1. Kaplan-Meier estimates for time to tuberculosis (TB) disease diagnosis not attributed to recent transmission among non–US-born persons stratified by age at entry to the United States, 2011–2018.

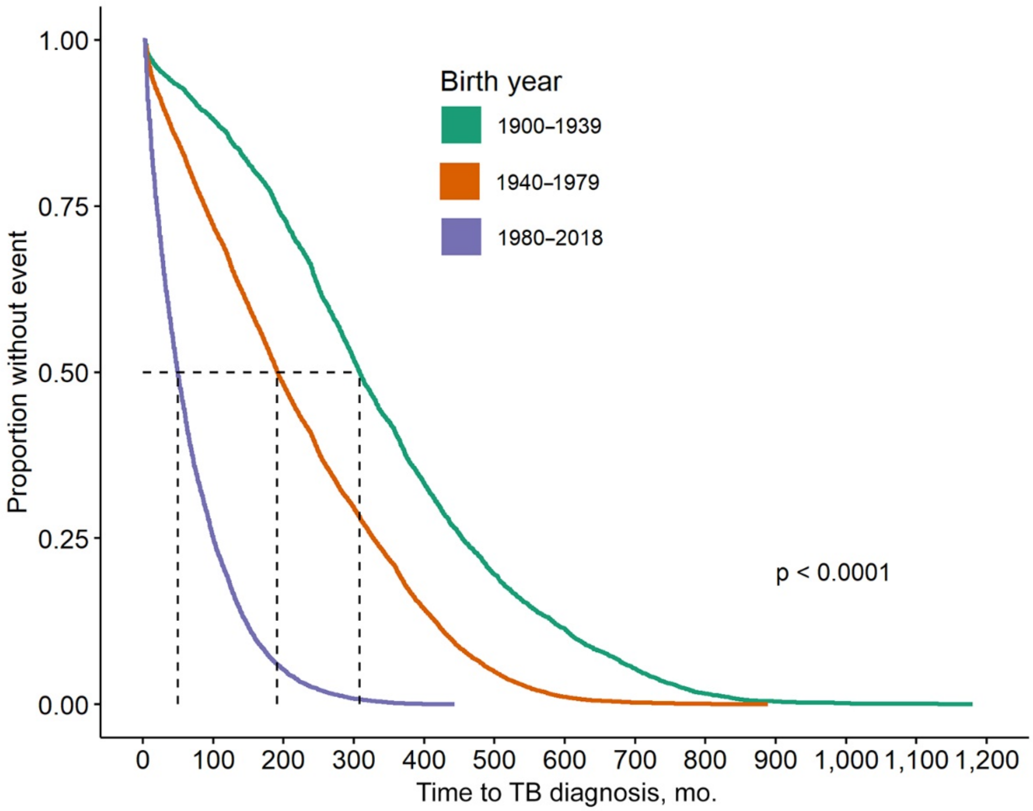

Appendix Figure 2. Kaplan-Meier estimates for time to tuberculosis (TB) disease diagnosis not attributed to recent transmission among non–US-born persons stratified by birth year, United States, 2011–2018.

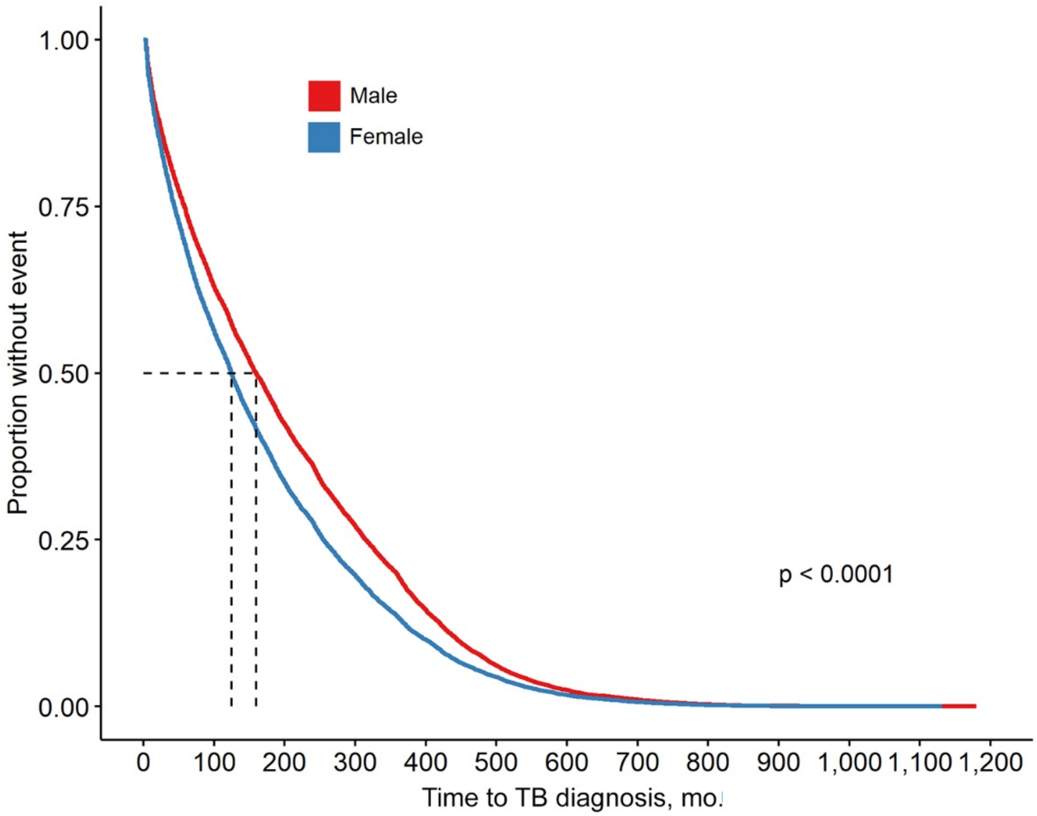

Appendix Figure 3. Kaplan-Meier estimates for time to tuberculosis (TB) disease diagnosis not attributed to recent transmission among non–US-born persons stratified by sex, United States, 2011–2018.
